# How Much Do You Fuse? A Comparison of Cell Fusion Assays in a Breast Cancer Model

**DOI:** 10.3390/ijms25115668

**Published:** 2024-05-23

**Authors:** Mareike Sieler, Jessica Dörnen, Thomas Dittmar

**Affiliations:** 1 Institute of Immunology, Center for Biomedical Education and Research (ZBAF), Witten/Herdecke University, Stockumer Str. 10, 58453 Witten, Germany; mareike.sieler@uni-wh.de (M.S.); jessica.doernen@ruhr-uni-bochum.de (J.D.); 2 Faculty of Medicine, Ruhr University Bochum, 44789 Bochum, Germany

**Keywords:** cell fusion, breast cancer, Syncytin-1, ASCT2, fluorescence-based cell fusion assays, quantification of cell fusion, dual split protein assay, fluorescence double reporter assay

## Abstract

Cell fusion is a biological process that is crucial for the development and homeostasis of different tissues, but it is also pathophysiologically associated with tumor progression and malignancy. The investigation of cell fusion processes is difficult because there is no standardized marker. Many studies therefore use different systems to observe and quantify cell fusion in vitro and in vivo. The comparability of the results must be critically questioned, because both the experimental procedure and the assays differ between studies. The comparability of the fluorescence-based fluorescence double reporter (FDR) and dual split protein (DSP) assay was investigated as part of this study, in which general conditions were kept largely constant. In order to be able to induce both a high and a low cell fusion rate, M13SV1 breast epithelial cells were modified with regard to the expression level of the fusogenic protein Syncytin-1 and its receptor ASCT2 and were co-cultivated for 72 h with different breast cancer cell lines. A high number of fused cells was found in co-cultures with Syncytin-1-overexpressing M13SV1 cells, but differences between the assays were also observed. This shows that the quantification of cell fusion events in particular is highly dependent on the assay selected, but the influence of fusogenic proteins can be visualized very well.

## 1. Introduction

Cell–cell fusion is an elementary biological mechanism that is crucial for various physiological processes, such as the fertilization of the egg cell, the formation of the placenta or the formation and homeostasis of muscles and bones [1,2,3,4,5]. However, cell fusion has also been linked to pathophysiological processes, such as the fusion of enveloped viruses with the host cell or in the development of various tumors [6,7,8,9]. Although it sounds like a simple process at first, cell fusion is very complex and strictly regulated. Several conditions and steps are required for the fusion of two or more cells, and the exact process is not yet fully understood [3,10].

What has been discovered to date is that the cells must first reach a fusogenic state, which is triggered by factors such as inflammation [11,12,13,14,15,16,17,18] or hypoxia [19,20,21], both of which are also found in the immediate vicinity of a tumor. The next step is the attachment of two or more pro-fusogenic cells to each other, which is catalyzed by so-called fusogenic proteins in order to overcome the energetic repulsion of the cells, followed by hemi fusion of the membranes, fusion pore opening and the complete fusion of the cells [4,22]. The time-dependent expression of these fusogenic proteins additionally regulates fusogenic triggers and cell fusion in an intrinsic manner, for example, during fertilization, the formation of the placenta or myogenesis [23]. One example of a fusogenic protein is the human endogenous retroviral (HERV) protein Syncytin-1, which can bind to alanine serine cysteine transporter 2 (ASCT2) on a neighboring cell and brings them in close proximity for fusion. The protein and the cell fusion mediated by it are essential for the formation of cytotrophoblasts out of trophoblasts and thus for the formation of the placenta during pregnancy [24,25,26,27,28,29]. The cell fusion taking place in the placenta is regulated by the time-dependent expression of Syncytin-1, which was detected in villous cytotrophoblasts until 37 weeks of gestation [30]. In addition, it was found that Syncytin-1 is also involved in the physiological fusion of myoblasts to form myotubes [31,32,33,34] and in the fusion of osteoclasts [35,36]. In contrast, the increased expression of Syncytin-1 was also detected in various tumors, such as non-small cell lung cancer [37,38], endometrial carcinoma [39,40,41] or breast cancer [42,43]. Syncytin-1′s role in both in the fusion of cancer cells with other cells and in the progression of a tumor disease was shown in various studies [13,37,39,40,42,43,44,45,46,47,48,49]. Fusogens in general, however, are expressed very heterogeneously in cancer cells, so that the range of measured cell fusion events is also quite large, both in in vivo studies with 0.0066–6.5% [50,51,52,53,54] and in in vitro studies with 0.5–51.0% [52,54,55,56,57,58,59]. Cancer hybrid cells resulting from such cell fusion events showed altered properties, like an enhanced metastatic potential, resistance to therapy or characteristics of cancer stem/initiating cells (CS/ICs) (for review, see [8,9,60,61,62]). The acquisition of new malignant properties of cancer hybrid cells makes cell fusion a therapeutic target in the fight against cancer.

Nevertheless, it is difficult to observe and quantify cell fusion events or to detect the resulting cancer hybrid cells. Cell fusion events that do not occur in an experimental setting can only be detected on the basis of the properties of the resulting hybrid cell, as there is no standardized cell fusion marker. For example, cancer hybrid cells were identified in tumors of female patients after a bone marrow transplant with a male donor using the X chromosome contained in the tumor cells, or DNA segments of the donor could be detected in cancer cells using short tandem repeat analysis [56,63,64]. Furthermore, cancer hybrid cells could be identified based on epitopes that are normally not found in cancer cells and are specific to macrophages or epithelial cells, for example [6,58,65,66,67,68]. 

In experimental studies, hybrid cells can be detected more easily by labeling the parental cells. These can be labeled with different fluorophores or labeled antibodies and probes, whereupon the resulting hybrid cells exhibit mixed fluorescence [56,69,70,71]. Furthermore, stimulated cells can be stained for their cell borders and nuclei, which then can be counted per cell to obtain the syncytial index [72,73,74,75,76]. Another approach is the usage of vectors coding for different antibiotic resistances on the parental cells, so that double selection of a coculture produces hybrid cells with both resistances [77,78,79,80]. However, studies should only be compared with caution, as they not only differ in their experimental design and the models and cancer types used but also use different methods for detecting cell fusion and hybrid cells, and the analyses were carried out at different time points. It is therefore possible that a later analysis time point does not necessarily reflect the cell fusion rate of the cancer cells but rather the proliferation of stable hybrid cells. 

In order to demonstrate the extent to which different cell fusion assays can actually differ from each other, two fluorescence-based cell fusion assays, namely the fluorescence double reporter (FDR) and dual split protein (DSP) assay, were used in this study under otherwise constant experimental conditions. Cell fusion between M13SV1 breast epithelial cells and the triple-negative breast cancer cell lines HS578T, MDA-MB-231 and MDA-MB-435S was observed after coculturing between wild-type cells with or without TNFα as inflammatory stimulus, but it was also induced by the overexpression of Syncytin-1 in the epithelial cells or inhibited by the knock-out (KO) of ASCT2. The obtained results clearly show that the quantification of cell fusion events differs greatly between the two assays used. However, the effect of overexpression of Syncytin-1 on the cell fusion rate could be clearly demonstrated in both assays. In conclusion, cell fusion assays are able to visualize the effect of fusogenic proteins or stimuli independently but show strong differences in the number of resulting hybrid cells.

## 2. Results

### 2.1. Evaluation of the Vectors for FDR and DSP Assay

**Figure 1:**
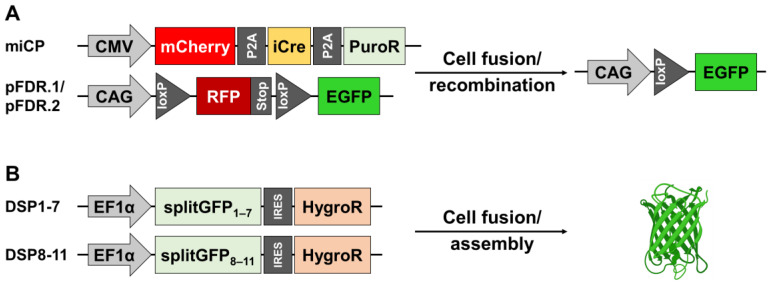
Functioning of the fluorescence double reporter (FDR) assay and the dual split protein (DSP) assay. (**A**) For the FDR assay, M13SV1 cell lines are transfected with an miCP vector, coding for a codon-optimized Cre recombinase (iCre), while the breast cancer cell lines stably express pFDR.1 or pFDR.2. The latter are recombined by iCre at the respective loxP sites during a cell fusion event, which leads to a change in fluorescence from red to green in the resulting hybrid cell. (**B**) For the DSP assay, the two cell lines to be fused each stably express a part of a split green fluorescent protein (GFP), which assemble to a functional GFP after cell fusion. Figure created with BioRender.

In order to quantify and compare the cell fusion rates of different cocultures of breast epithelial cells and breast cancer cells, two fluorescence-based cell fusion assays were used. The FDR assay is based on the principle that one cell line to be fused expresses a vector coding for a Cre recombinase (miCP) and the other cell line to be fused expresses a pFDR vector coding for a red fluorescent protein (RFP) flanked by two loxP sites (Figure 1A) [16]. After a cell fusion event has taken place, the pFDR vector is recombined by Cre, resulting in a fluorescence switch from red to green. The DSP assay is based on the principle that both cell lines to be fused express different split green fluorescent protein (GFP) subunits, which, after a cell fusion event, form a functional GFP (Figure 1B). The two fluorescence-based cell fusion assays were chosen due their different ways in which the fluorescence of a hybrid cell changes or develops.

**Figure 2:**
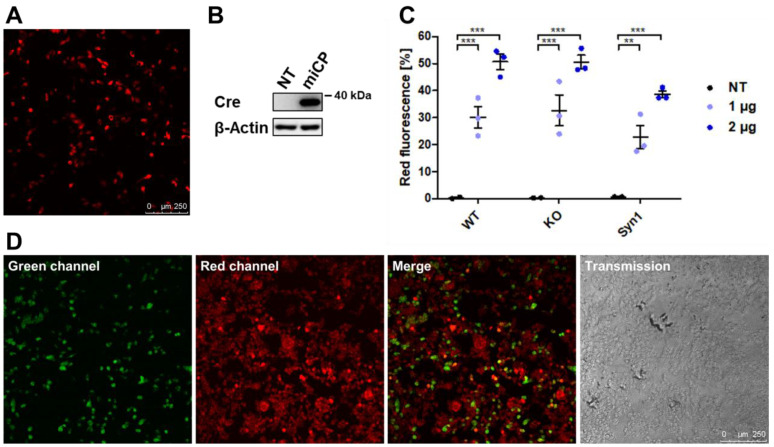
Characterization of the functionality of the miCP vector. (**A**) The confocal laser scanning microscopy picture shows the red fluorescence of the mCherry fluorescent protein in M13SV1 cells transfected with miCP. Bar = 250 µm. (**B**) Western blot data indicate the overexpression of iCre recombinase 48 h after transfection in M13SV1 cells. (**C**) Flow cytometry data show different percentages of red fluorescent cells in the different M13SV1 cell lines 48 h after transfection with 1 µg or 2 µg of miCP vector. It is noticeable that the transfection of more vector leads to more uniform results, but the M13SV1_Syn1 cells contain the fewest red fluorescent cells. The flow cytometry measurements were not performed at the emission optimum of mCherry, so the actual number of fluorescent cells could be higher. NT = non-transfected control. ** = *p* ≤ 0.01; *** = *p* ≤ 0.001. (**D**) Confocal laser scanning microscopy pictures of MDA-MB-435S_pFDR.1 cells transfected with miCP show green fluorescent cells 48 h after transfection and thus the functionality of the iCre and the loxP vector system. Bar = 250 µm.

In order to improve the efficiency of the FDR assay, we designed a Cre expression vector which encodes the sequence mCherry-P2A-iCre-P2A-PuroR and can therefore be used for the expression of a codon-optimized Cre recombinase (iCre) under puromycin selection and simultaneous mCherry expression control. In order to test the functionality of the vector, after transfection in M13SV1 cells, the red fluorescence caused by mCherry expression was first examined microscopically (Figure 2A), and the expression of iCre was detected by means of a Western blot (Figure 2B). A stable expression of the vector in the cells under puromycin selection was not possible, as the continuous expression of iCre turned out to be cytotoxic. Therefore, the cells for the FDR assay were freshly transfected with the vector, and the amount of red fluorescent cells expressing mCherry was determined using flow cytometry, which showed that the transfection of more plasmid DNA led to more fluorescent cells (Figure 2C). In order to test not only the expression but also the functionality of iCre, MDA-MB-435S_pFDR.1 cells were transfected with miCP and examined microscopically (Figure 2D). The confocal laser scanning microscopy pictures show, in addition to red fluorescent cells, a large number of green fluorescent cells, which indicates the correct function of iCre coded on the miCP vector. 

**Figure 3:**
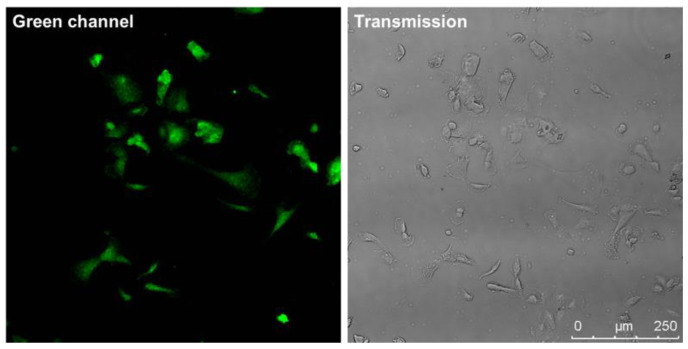
Confocal laser scanning microscopy pictures of M13SV1 cells co-transfected with DSP1-7 and DSP8-11 vectors show the green fluorescence of the cells and thus the functionality of the vectors. Bar = 250 µm.

The functionality of the DSP vectors was examined via the co-transfection of both the DSP1-7 and DSP8-11 vectors into M13SV1 cells and the selection of transfected cells with hygromycin. The confocal laser scanning microscopy picture shows green fluorescent cells, which indicates the correct function and ability to assemble of the splitGFPs (Figure 3). Cells that do not show green fluorescence after selection with hygromycin have probably taken up only one of the two co-transfected vectors.

### 2.2. Successful KO of ASCT2 and Overexpression of Syncytin-1 in M13SV1 Cells

**Figure 4:**
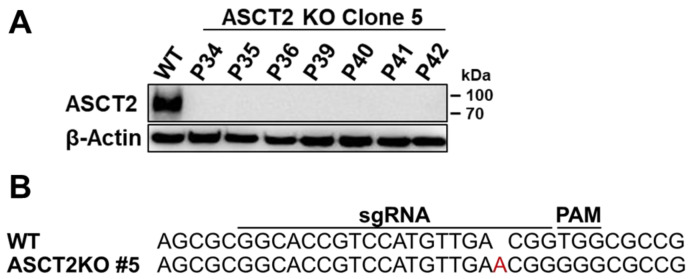
ASCT2 was successfully knocked-out in M13SV1 cells. (**A**) Western blot data of several passages (P) of the isolated single cell clone 5 bearing the stable ASCT2 KO. (**B**) Sequencing data indicate a nucleotide insertion (red) in M13SV1_ASCT2KO clone 5 cells.

Cell fusion events in the context of this study should be induced by the interaction of Syncytin-1 with its receptor ASCT2. For a fusion-incompetent cell population, an M13SV1 cell line carrying a KO for ASCT2 was created using CRISPR/Cas9 (M13SV1_ASCT2KO). Single cell clone 5 was tested for the stability of the ASCT2 KO by collecting samples over several passages (P) for analysis with Western blotting. Figure 4A shows the consistency of the ASCT2 KO for clone 5 from passage 34 up to 42, which corresponds to several weeks of cultivation and thus confirms a stable KO. Genomic DNA of ASCT2 KO clone 5 was isolated and sequenced at the CRISPR/CAS9 mutation site and bears an insertion at the Cas9 cutting site, leading to the loss of ASCT2 expression (Figure 4B).

**Figure 5:**
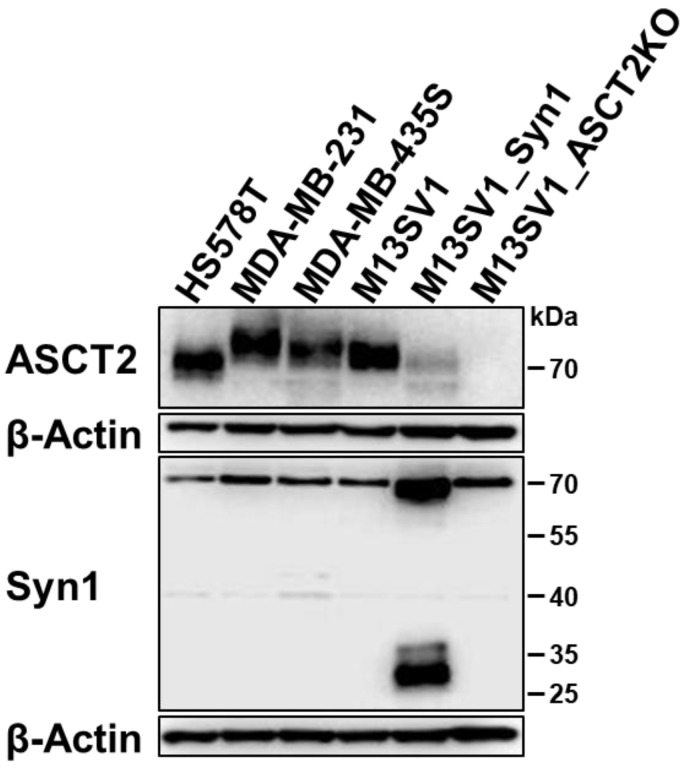
Expression levels of ASCT2 and Syncytin-1 in M13SV1 cell lines and breast cancer cell lines used for cell fusion assays.

To induce Syncytin-1-medited cell fusion to a high degree in the cocultures, a M13SV1 cell line was also generated that stably overexpresses Syncytin-1 by transfecting a vector coding for a Syncytin-1-P2A-PuroR sequence (M13SV1_Syn1). The overexpression of Syncytin-1 was confirmed with Western blot analysis and shows the premature and post-translationally modified protein at 73 kDa as well as one part of the active form of Syncytin-1 cut by furin protease between 25 and 35 kDa (gp24 subunit), which is detected by the used antibody (Figure 5). It is also noticeable that ASCT2 expression is greatly reduced in M13SV1_Syn1 cells compared to wild-type M13SV1 cells. However, the ASCT2 KO in M13SV1 cells had no effect on the Syncytin-1 expression level. Furthermore, it can be seen that all breast cancer cell lines exhibit strong ASCT2 expression as well as basal Syncytin-1 expression of the premature protein. The size of ASCT2 in the Western blot varies slightly due to different glycosylation levels of the protein between the cell lines. All cell lines shown in Figure 5 were further used for cell fusion experiments.

### 2.3. Homotypic DSP Cell Fusion Assay

**Figure 6:**
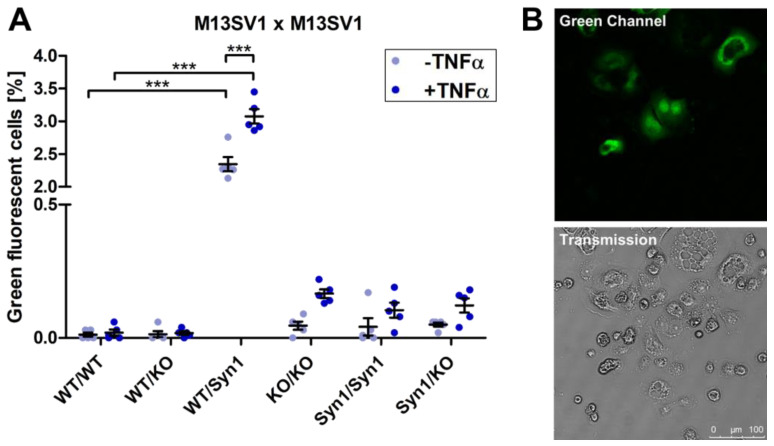
The measurement of cell fusion with the DSP assay in homotypic cocultures of M13SV1 cell lines. (**A**) M13SV1 cells with different expression levels of Syncytin-1 and ASCT2 were cocultured in a 1:1 ratio for 72 h with or without the addition of 100 ng/mL TNFα. The amount of green fluorescent cells was measured via flow cytometry. Shown are the means ± S.E.M. of five independent experiments. Statistical significance was calculated using a one-way ANOVA and Bonferroni’s multiple comparison test. *** = *p* ≤ 0.001. (**B**) Representative confocal laser scanning microscopy pictures show the green fluorescence of hybrid cells of the homotypic coculture of M13SV1_DSP1-7 wild-type cells and M13SV1_Syn1_DSP8-11 cells. The cells were cocultured in a 1:1 ratio on a chamber slide for 72 h and fixed for microscopy studies. Bar = 100 µm.

A homotypic cell fusion assay was set up with the three M13SV1 cell lines expressing the corresponding DSP vectors and with differently altered ASCT2 and Syncytin-1 expression levels by coculturing them in a ratio of 1:1 for 72 h with or without the inflammatory cytokine TNFα as stimulus for cell fusion, with subsequent analysis of the cell fusion with flow cytometry and microscopy (Figure 6). In flow cytometry, cocultures of M13SV1_DSP1-7/DSP8-11 wild-type cells (WT/WT) or of M13SV1_DSP1-7 wild-type cells with M13SV1_ASCT2KO_DSP8-11 cells (WT/KO) showed no fusogenic activity both with and without TNFα (Figure 6A). In contrast, the coculture of M13SV1_DSP1-7 wild-type cells with M13SV1_Syn1_DSP8-11 cells resulted in a significantly increased amount of green fluorescent cells (2.4 ± 0.1%), which was further significantly increased through the addition of TNFα in the coculture (3.1 ± 0.1%). In the cocultures of M13SV1_ASCT2KO_DSP1-7/DSP8-11 cell lines (KO/KO) or M13SV1_Syn1_DSP1-7/DSP8-11 cells (Syn1/Syn1) or the combination of both (M13SV1_Syn1_DSP1-7/M13SV1_ASCT2KO_DSP8-11; Syn1/KO), only low amounts of green fluorescent cells could be detected. In these cocultures, a slight increase in the cell fusion rate was observed with the addition of TNFα, which may indicate another possible fusion process independent of Syncytin-1 and ASCT2, which does not occur in wild-type cells. The high amount of green fluorescent cells in cocultures of M13SV1_DSP1-7 wild-type and M13SV1_Syn1_DSP8-11 cells (WT/Syn1) could also be detected via confocal laser scanning microscopy (Figure 6B). In addition to green fluorescent hybrid cells, unfused cells can also be seen in images taken in transmitted light.

### 2.4. Heterotypic DSP Cell Fusion Assay

**Figure 7:**
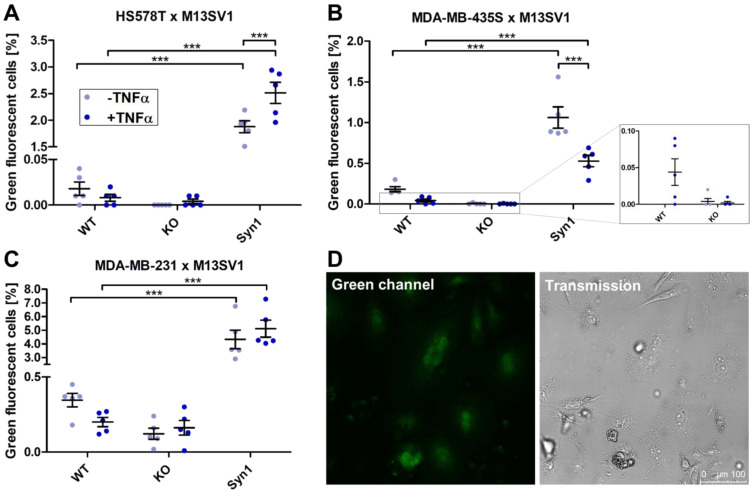
Measurement of cell fusion with the DSP assay in cocultures of M13SV1 cell lines expressing DSP8-11 with HS578T_DSP1-7, MDA-MB-435S_DSP1-7 and MDA-MB-231_DSP1-7 breast cancer cell lines. M13SV1_DSP8-11 cells with different expression levels of Syncytin-1 and ASCT2 were cocultured with (**A**) HS578T_DSP1-7 cells, (**B**) MDA-MB-435S_DSP1-7 cells or (**C**) MDA-MB-231_DSP1-7 cells in a 1:3 ratio for 72 h with or without the addition of 100 ng/mL TNFα. The amount of green fluorescent cells was measured by flow cytometry. Shown are the means ± S.E.M. of five independent experiments. Statistical significance was calculated using a one-way ANOVA and Bonferroni’s multiple comparison test. *** = *p* ≤ 0.001. (**D**) Representative confocal laser scanning microscopy pictures show the green fluorescence of hybrid cells of the coculture of M13SV1_Syn1_DSP8-11 cells with MDA-MB-231_DSP1-7 cells. The cells were cocultured in a 1:3 ratio on a chamber slide for 72 h and fixed for microscopy studies. Bar = 100 µm.

A heterotypic cell fusion assay was set up with the three different M13SV1_DSP8-11 cell lines (wild type, ASCT2KO, Syn1 overexpression) and three breast cancer cell lines (MDA-MB-231, MDA-MB-435S and HS578T) expressing the DSP1-7 vector in a ratio of 1:3 for 72 h with or without the inflammatory cytokine TNFα as stimulus for cell fusion, with subsequent analysis of the cell fusion with flow cytometry and microscopy (Figure 7). 

In flow cytometry, cocultures of the breast cancer cell lines with M13SV1_DSP8-11 wild-type or M13SV1_ASCT2KO_DSP8-11 cells yielded in zero to low amounts of green fluorescent cells, which were not significantly altered by addition of TNFα (Figure 7A–C). Cocultures of M13SV1_Syn1_DSP8-11 cells with the breast cancer cells expressing DSP1-7 yielded the highest amount of green fluorescent cells, which differs significantly from the amount of other cocultures. The coculture of MDA-MB-231_DSP1-7 cells showed the highest value of fluorescent cells (4.3 ± 0.7%), which could be further increased by the addition of TNFα (5.1 ± 0.6%) (Figure 7C). Representative confocal laser scanning microscopy pictures of the resulting hybrid cells, which appear to be polyploid, are shown in Figure 7D. The coculture of HS578T_DSP8-11 cells with M13SV1_Syn1_DSP1-7 cells also showed a high number of green hybrid cells (1.9 ± 0.1%), which could also be increased by the addition of TNFα (2.5 ± 0.2%) (Figure 7A). Although the highest value of fluorescent cells was also detected in the coculture of M13SV1_Syn1_DSP1-7 cells with MDA-MB-435S_DSP8-11 cells (1.1 ± 0.1%), this was reduced by the addition of TNFα (0.5 ± 0.1%) (Figure 7B).

### 2.5. Heterotypic FDR Cell Fusion Assay

**Figure 8:**
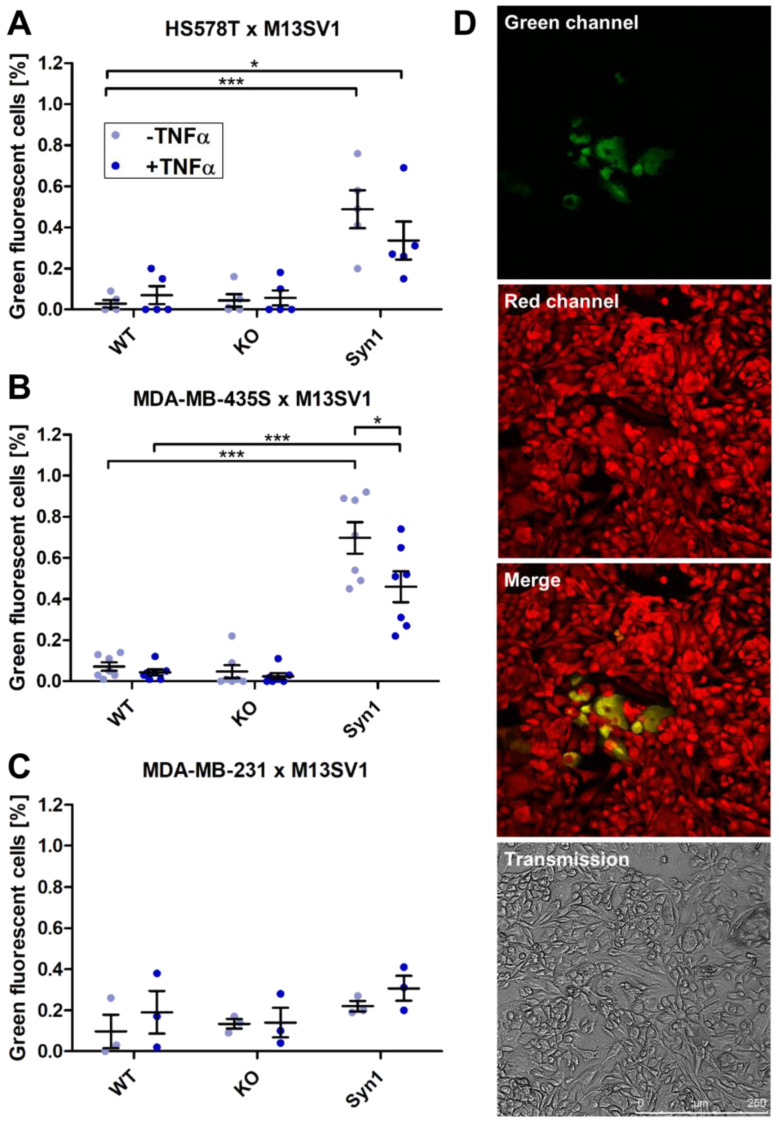
Measurement of cell fusion with the FDR assay in cocultures of M13SV1_miCP cell lines with breast cancer cell lines HS578T_pFDR.2, MDA-MB-435S_pFDR.1 and MDA-MB-231_pFDR.2. M13SV1_miCP cells with different expression levels of Syncytin-1 and ASCT2 were cocultured with (**A**) HS578T_pFDR.2 cells, (**B**) MDA-MB-435S_pFDR.1 cells or (**C**) MDA-MB-231_pFDR.2 cells in a 1:3 ratio for 72 h with or without the addition of 100 ng/mL TNFα. The amount of green fluorescent cells was measured by flow cytometry. Shown are the means ± S.E.M. of three to seven independent experiments. Statistical significance was calculated using a one-way ANOVA and Bonferroni’s multiple comparison test. * = *p* ≤ 0.05; *** = *p* ≤ 0.001. (**D**) Representative confocal laser scanning microscopy pictures show the green fluorescence of hybrid cells of the coculture of M13SV1_Syn1_miCP cells with MDA-MB-435S_pFDR.1 cells. The cells were cocultured in a 1:3 ratio on a chamber slide for 72 h and fixed for microscopy studies. Bar = 250 µm.

A heterotypic cell fusion assay was set up with the three M13SV1 cell lines (wild type, ASCT2KO, Syn1 overexpression), which were freshly transfected with an miCP vector coding for the iCre recombinase and the three breast cancer cell lines MDA-MB-231_pFDR.2, MDA-MB-435S_pFDR.1 and HS578_pFDR.2. The coculture was seeded in a ratio of 1:3 for 72 h with or without the inflammatory cytokine TNFα as stimulus for cell fusion, with subsequent analysis of the cell fusion rate with flow cytometry and microscopy (Figure 8).

In flow cytometry, cocultures of the breast cancer cell lines HS578T_pDFR.2 and MDA-MB-435S_pFDR.1 with M13SV1_miCP wild-type or M13SV1_ASCT2KO_ miCP cells yielded low amounts of green fluorescent cells beneath 0.1%, which were not significantly altered by addition of TNFα (Figure 8A,B). Cocultures of M13SV1_Syn1_miCP cells with these two breast cancer cell lines yielded the highest amount of green fluorescent cells, which differed significantly from the amount of other cocultures. The coculture of HS578T_pDFR.2 cells showed 0.49 ± 0.09% fluorescent cells, which was decreased by the addition of TNFα (0.34 ± 0.09%) (Figure 8A). A similar result was obtained for the coculture with MDA-MB-435S_pFDR.1 cells with 0.70 ± 0.08% green fluorescent cells without TNFα, which, through the addition of cytokine, was decreased to 0.46 ± 0.08% (Figure 8B). Representative confocal laser scanning microscopy pictures of the cells, which appear to be polyploid, are shown in Figure 8D. The cocultures of MDA-MB-231_pFDR.2 cells with M13SV1_miCP cell lines showed no significant differences in the amount of green fluorescent cells and were all comparably low (Figure 8C). The examination of the MDA-MB-231_pFDR.2 cells by direct transfection with miCP resulted in very few detectable green fluorescent cells, from which it can be concluded that the function of this cell line for the fusion assays was not given.

## 3. Discussion

Cell fusion is an essential process for the development and maintenance of living organisms. Some details of the process have been elucidated in recent years, but much still remains unknown. For example, why and how are cancer cells able to fuse with other cells? However, since the resulting cancer hybrid cells may exhibit new malignant properties, precise research into cell fusion is essential, particularly in the context of tumors. As no general fusion marker exits to date, scientists use a wide variety of methods to detect cell fusion. The extent to which the experiments and their results can deviate from each other and thus be misinterpreted was to be shown in this study. 

**Figure 9:**
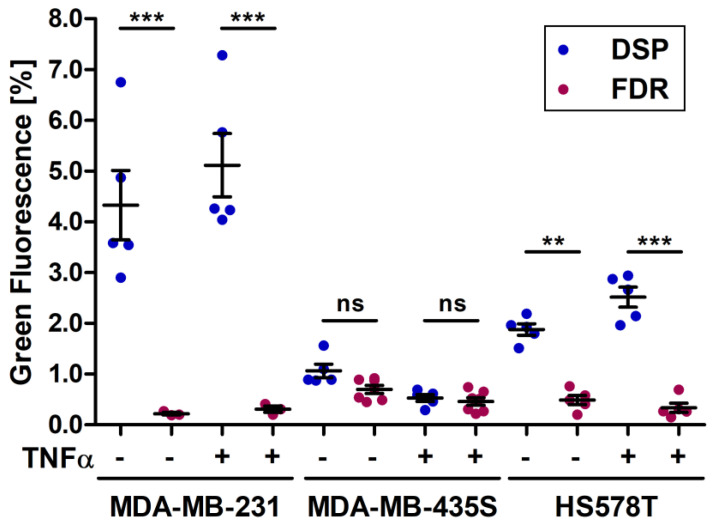
Comparison of the amount of hybrid cells detected in cocultures of M13SV1_Syn1 cells with the breast cancer cell lines MDA-MB-231, MDA-MB-435S and HS578T either in the DSP assay or in the FDR assay measured using green fluorescent cells. The cocultures were set up in a 1:3 ratio for 72 h with (+) or without (−) the addition of 100 ng/mL TNFα. The amount of green fluorescent cells was measured by flow cytometry. Shown are the means ± S.E.M. of three to seven independent experiments. Statistical significance was calculated using a one-way ANOVA and Bonferroni’s multiple comparison test. ns = not significant; ** = *p* ≤ 0.01; *** = *p* ≤ 0.001.

By keeping stable as many assay components as possible, such as identical cell lines, passages, cultivation conditions, assay performance and readout, it could be shown that the detectable cell fusion rate can differ significantly between the assays. While the cocultures of M13SV1 wild-type cells or M13SV1_ASCT2KO cells with the breast cancer cell lines showed comparable results between the two assays, probably due to the generally low fusogenicity of these cells, the cocultures of breast cancer cells with M13SV1_Syn1 cells showed strong differences between the assays (Figure 9).

The comparison of the amount of green fluorescent cells detected showed significant differences between the DSP assay and the FDR assay for cocultures of M13SV1_Syn1 cells and MDA-MB-231 cells and HS578T cells, while, for cocultures with MDA-MB-435S cells, no significant differences in the results of the assays could be seen. The significant differences between the assays can be explained by several aspects, which are discussed below. 

### 3.1. Impact of the Chosen Model Organisms on Cell Fusion Experiments

In this study, M13SV1 breast epithelial cells were modified with respect to their expression level of Syncytin-1 and its receptor ASCT2, and cocultures were set up with the three breast cancer cell lines MDA-MB-231, MDA-MB-435S and HS578T. Two important observations were made when looking at the expression levels of Syncytin-1 and ASCT2 in all the cell lines used. This is, on the one hand, the downregulation of ASCT2 in the M13SV1_Syn1 cells, which is probably due to an effect called superinfection resistance, which has already been observed with Syncytin-1 and other retroviral elements that downregulate their own receptor after infection of a cell in order to prevent infection by another, related virus [81,82,83,84]. On the other hand, the Western blot shows the presence of the active, cleaved form of Syncytin-1 in these cells, which could not be found in any of the other cell lines.

With these observations in mind, the results of the homogenous DSP assay between the different M13SV1 cell lines, which only showed a high cell fusion rate in the coculture of M13SV1_Syn1 cells with M13SV1 wild-type cells, can be explained (Figure 6). Syncytin-1-mediated cell fusion can only function with cells that express ASCT2, which is not the case with M13SV1_ASCT2KO and also not with M13SV1_Syn1 cells (Figure 5). As expected, the fusion rate was increased through the addition of TNFα to the cocultures, as inflammatory mediators are known to induce cell fusion [11,15,16,85,86]. The lack of fusion between the other cell lines can be explained by the fact that Syncytin-1 is not present in the active state or possibly is not localized in the plasma membrane, where it must be localized to induce cell fusion. Another hypothesis as to why fusion between the cells may fail to occur has been proposed, which states that a certain balance between ASCT2 and Syncytin-1 expression must exist between neighboring cells in order to trigger cell fusion [28]. In addition to inducing cell fusion, the overexpression of Syncytin-1 can also lead to increased pinching of extracellular vesicles [87], which may contain assay components and can be taken up by other cells, leading to false positive signals in the assay. Another observation in the results of the homotypic cell fusion assay is the slight increase in the number of green fluorescent cells after the addition of TNFα in cocultures with cells that do not express ASCT2, which could be explained by the possible activation of proteins that have an influence in cell fusion, such as membrane-bound scramblases [76,88]. 

In the heterotypic fusion assays, differences in the amounts of green fluorescent cells when using the same passage M13SV1_Syn1 may not be due to different expression levels of ASCT2, since the breast cancer cell lines all express a high amount of the protein. In general, it is assumed that heterogeneous cell fusion between cancer cells and normal cells, both of which are in a fusogenic state, occurs more frequently than homogenous cell fusion of the same cells [23]. For example, this was observed by Melzer et al. in the significantly lower occurrence of homofusion of breast cancer cells compared to heterofusion of breast cancer cells with mesenchymal stem cells [11]. In the results obtained here, however, only the heteroculture of M13SV1_Syn1 cells with MDA-MB-231 cells showed a significantly higher proportion of green fluorescent cells than the homoculture of M13SV1 wild-type cells with M13SV1_Syn1 cells in the DSP assay. The cocultures of M13SV1_Syn1 cells with MDA-MB-231 or HS578T cells have in common that stimulation with TNFα lead to an increase in the cell fusion rate, as expected. However, in the coculture of MDA-MB-435S cells, TNFα stimulation led to a reduction in the proportion of green fluorescent cells, which could possibly be due to the sensitivity of the cells to TNFα [89]. An additional point of criticism of this cell lines is the unresolved question of whether it originates from a ductal carcinoma or a melanoma [90,91,92,93]. The parental cell line MDA-MB-435 shows the expression of melanocytic genes, but it is also able to produce milk proteins after differentiation [94]. Possible causes for these properties are a lineage fidelity after differentiation of the cells or a contamination of the original breast cancer cells with a melanoma cell line [94]. These differences between the triple-negative breast cancer cell lines MDA-MB-231 and HS578T, which are very similar in their characteristics [95], could be a reason for the deviating behavior of MDA-MB-435S cells on TNFα. 

### 3.2. What to Look out for When Evaluating Cell Fusion Assays

The large differences between the detected fusion cells may not only be due to the different cell lines used but also to the function of the DSP assay and FDR assay. Although stable cell lines could be generated with the DSP vectors in which the split-GFP gene is directly linked to hygromycin resistance via an IRES sequence, it is possible that not all resistant cells express the split-GFP. In a study by Lanza et al., in which the efficiency of various antibiotic resistances in HT1080 or HEK293 cells was tested using a vector with a GFP-IRES selection marker sequence, it was seen that the vector with hygromycin resistance led to green fluorescence in 11 of 14 cell clones (79%) [96]. In addition, a study on the efficiency of antibiotic resistances in multicistronic vectors by Guo et al. revealed that hygromycin performed rather mediocrely as antibiotic selection marker [97]. However, the experiments were always carried out with the same generated cell lines so that the results remain comparable within a coculture test series.

While the splitGFP constructs of the DSP assay are non-toxic to cells and can be continuously expressed, the permanent expression of the miCP vector turned out to be problematic due to the high activity of the iCre recombinase. Although this has been the aim of the vector design, it was only subsequently realized that iCre cuts DNA unspecifically without existing recognition sequences, which led to the initiation of apoptosis in the cells [98,99,100,101,102]. The different M13SV1 cell lines were therefore freshly transfected with miCP before each FDR cell fusion assay, with the M13SV1_Syn1 cells expressing the vector less strongly than the WT cells and the ASCT2 KO cells (Figure 2C). The transfection itself and the variability of the transfection efficiency between the cell lines entail both a greatly increased effort for the FDR assay and less good reproducibility of the experiments. The unintended induction of apoptosis through iCre expression could have an influence on cell fusion with breast cancer cells, since a feature of early apoptosis is the scramblase-mediated externalization of phosphatidylserine to the surface of the cell membrane, where it can be recognized by Annexin V and induce the fusion of two cells [76,103]. For example, it is known that Annexin V plays a role in the fusion of myoblasts, osteoclasts and trophoblasts [104]. In a study by Noubissi et al., it was also observed that the addition of apoptotic cells to a coculture of mesenchymal stem cells and human breast cancer cells induces cell fusion [105]. Similar observations were made in a study by Hochreiter-Hufford et al., which investigated the influence of apoptotic cells on the fusion of myoblasts [106]. While it was shown in this study that apoptotic cells do not fuse directly with other cells but merely induce the fusion of non-apoptotic cells, this was not investigated in more detail in the study of Noubissi and colleagues.

In this context, it can also be discussed why the cocultures of HS578T cells with M13SV1_Syn1 cells showed an increase in green fluorescent cells in the DSP assay with TNFα but a decrease in the FDR assay. It is possible that the HS578T cells in this assay reacted to a combination of TNFα and the FDR assay components. In general, far fewer hybrid cells were detected in the FDR assay than in the DSP assay, which may, among other things, be related to suboptimally designed pFDR vectors. Firstly, in case of the pFDR.1 vector, its antibiotic resistance gene is not directly linked to the expression of the LoxP-flanked fluorophore gene, which can lead to the loss of the expression of this cassette. While pFDR.1 encodes the gene for puromycin resistance, pFDR.2 is a neomycin resistance gene, which is one of the weaker selection markers [96,97]. In coculture experiments with MDA-MB-231 cells in the FDR assay, hardly any fluorescence could be detected, just as no effect of Syncytin-1 overexpression on the cell fusion rate could be observed, which is probably not due to non-occurring cell fusion (see results DSP assay) but rather to the inadequate expression of the pFDR.2 vector. The lower efficiency of the vector is also reflected in the lower amount of detected fusion cells from the HS578T_pFDR.2 cells cocultured with M13SV1_Syn1_miCP cells in comparison to the DSP assay. In contrast, the pFDR.1 vector leads to robust red fluorescence in MDA-MB-435S cells under puromycin selection due to the expression of DsRed2, which can switch to green when Cre recombinase is introduced into the cells. The working group has already tried to introduce the pFDR.1 vector into the other breast cancer cell lines, but this led to random green fluorescent cells in HS578T cells. Presumably, the genomic instability of the cells leads to uncontrolled recombination of the vector DNA, so that these cells would not be suitable for a fusion assay. In addition, these observations lead to the idea that the cells are also capable of modifying the pFDR.2 vector independently and can, for example, cut out the gene for EGFP. This would lead to a lower number of detectable cell fusion events in the FDR assay and thus to false negative results, which could explain the low cell fusion rate in the FDR assay in comparison to the DSP assay. Another reason for the reduced functionality of the FDR assay could be that the pFDR.2 vector was virally transduced into the cells and thus stably incorporated into the genome of the cells, which is why its recombination is also dependent on the accessibility of the gene sequence for the Cre recombinase. 

A disadvantage both assays have in common is that the fusion of already-fused cells with other cells cannot be displayed, as this cannot be distinguished from a signal from only two fused cells. Therefore, it is possible that more cell fusion events have taken place but were not detected by flow cytometry. In addition, the fusion between two cells with the same assay component cannot be displayed, as this does not result in a detectable signal. This is a particular problem with homotypic cocultures in which the cell lines have not been modified or have been modified equally, like, for example, in the coculture of M13SV1 wild-type cells in the DSP assay. With regard to the observation of the kinetics of fusion processes, the FDR assay presumably results in a longer delay of the signal than with the DSP assay. After fusion of the cells, the iCre recombinase must first detect and recombine the LoxP sites of the FDR vector so that the gene for GFP can be expressed. Compared to the simple combination of two already-expressed splitGFPs in the DSP assay, this process takes longer.

In summary, the cell fusion assays are suited to determine the role of a fusion protein or reagent in the fusion between one or more cell lines, whereby the DSP assay is better than the FDR assay in terms of handling and composition of the assay components. The experiments we performed show clear differences between the amounts of green fluorescent hybrid cells detected that cannot occur by chance and are due to the modifications of the M13SV1 cell lines. On the other hand, it could be shown that strong differences between the assays can exist under otherwise constant experimental conditions, which must be taken into account in the future planning of experiments and also in the collection of literature.

## 4. Materials and Methods

### 4.1. Cell Culture

M13SV1 human breast epithelial cells (kind gift of James Trosko, Michigan State University, East Lansing, MI, USA [107]), HS578T breast cancer cells (HTB 126; LGC Standards GmbH, Wesel, Germany), MDA-MB-231 breast cancer cells (HTB-26; LGC Standards GmbH, Wesel, Germany) and MDA-MB-435S breast cancer cells (HTB-129; LGC Standards GmbH, Wesel, Germany) were maintained in standard media (MDA-MB-435S and MDA-MB-231: DMEM; HS578T and M13SV1: RPMI 1640) (PAN Biotech GmbH, Aidenbach, Germany) supplemented with 10 % fetal calf serum (FCS; PAN Biotech GmbH, Aidenbach, Germany) and 100 U/mL penicillin/0.1 mg/mL streptomycin (PenStrep; PAN Biotech GmbH, Aidenbach, Germany). The following supplements were further added to the culture medium: M13SV1—10µg/mL recombinant human epidermal growth factor (rhEGF), 5 µg/mL human recombinant insulin, 0.5 µg/mL hydrocortisone, 4 µg/mL human transferrin and 10 nM β-estrogen (all supplements were purchased from Merck KGaA, Darmstadt, Germany). 

Nucleofection of the breast cancer cell lines MDA-MB-231 and HS578T with the pFDR.2 vector is described elsewhere, as well as the lentiviral transduction of MDA-MB-435S with the pFDR.1 vector [16]. HS578T_pFDR.2 and MDA-MB-231_pFDR.2 cells were maintained in media containing 400 µg/mL G418 (Merck KGaA, Darmstadt, Germany) and MDA-MB-435S_pFDR.1 cells in media containing 2 µg/mL puromycin (Thermo Fisher Scientific, Wesel, Germany). 

M13SV1 cells stably expressing pcDNA3.1_Syncytin-1-P2A-PuroR (see Section 2.3) were maintained in medium with 2 µg/mL puromycin (Thermo Fisher Scientific, Wesel, Germany). 

Cell lines stably expressing DSP1-7 or DSP8-11 vector (see Section 2.4) were maintained in their respective medium with additional 200 µg/mL Hygromycin B (Pan Biotech, Aidenbach, Germany).

All cells were cultivated in a humidified atmosphere at 37 °C and 5 % CO_2_.

### 4.2. Generation of ASCT Knock-Out (KO) M13SV1 Cells

M13SV1 cells bearing a KO of ASCT2 were generated by CRISPR/Cas9. The guide RNA sequence (5′-GGC ACC GTC CAT GTT GAC GG-3′) was determined using the E-CRISP website (www.e-crisp.org; accessed on 22 October 2019) [108]. Sense and antisense oligonucleotides (ASCT2_11_Fwd: 5′-CAC CGG CAC CGT CCA TGT TGA CGG-3′, ASCT2_11_Rev: 5′-AAA CCC GTC AAC ATG GAC GGT GCC-3′; Thermo Fisher Scientific, Wesel, Germany) were annealed and ligated into the BbSI digested pX330-U6-Chimeric_BB-CBh-hSpCas9-P2A-PuroR plasmid, the cloning of which is described elsewhere [109]. The pX330-U6-Chimeric_BB-CBh-hSpCas9-P2A-PuroR_ASCT2_11 plasmid was amplified in DH5α competent bacteria (Thermo Fisher Scientific, Wesel, Germany) and purified using the Nucleospin^®^ Plasmid Transfection-grade kit in accordance with the manufacturer’s instructions (Macherey-Nagel GmbH, Düren, Germany). Cloning was verified by Sanger Sequencing (Eurofins Genomics, Ebersbach, Germany). The analysis of sequencing data was performed using SnapGene 5.3.1 software (Dotmatics, Boston, MA, USA).

The M13SV1 cells were transfected with pX330-U6-Chimeric_BB-CBh-hSpCas9-P2A-PuroR_ASCT2_11 (1 µg) using the jetOPTIMUS^®^ DNA transfection reagent as recommended by the manufacturer (Polyplus, Illkirch, France). To select transfected cells from non-transfected cells, 2 µg/mL puromycin (Thermo Fisher Scientific, Wesel, Germany) was added to the culture medium 24 h after transfection for 48 h. Dead cells were removed by washing with phosphate-buffered saline (PBS). For the isolation of single cell clones, 100–200 cells were seeded in a Ø 100 mm cell culture dish. Single cell clones were picked using 3.2 mm cloning discs (SP Bel-Art/Behr Labor-Technik GmbH, Düsseldorf, Germany), transferred to 24-well plates and propagated. Growing clones were transferred to bigger cell culture flasks once they reached confluence.

The successful KO of ASCT2 was validated by Sanger Sequencing. Therefore, genomic DNA of the KO cell lines was isolated using the NucleoSpin^®^ Tissue kit in accordance with the manufacturer’s instructions (Macherey-Nagel GmbH, Düren, Germany). First, the CRISPR/Cas9 edited gene fragment was amplified by PCR (T7E1_ASCT2_Potter_fwd: 5′-GTG ACA GGG CTT TGC TTA GG-3′; T7E1_ASCT2_Potter_rev: 5′-TCT CCA GAC CCA CAC TCA CA-3′; primers as published by Potter et al. [110]), using Q5 Hot Start High-Fidelity DNA Polymerase (New England Biolabs, Frankfurt am Main, Germany), and then Sanger Sequencing (Eurofins Genomics, Ebersbach. Germany) was performed, using the primer Seq_ASCT2_11 (5′-AGA AGG TGG GAG GTT AGG CAG ATG-3′). The analysis of sequencing data was carried out using SnapGene 5.3.1 software (Dotmatics, Boston, MA, USA). Only cells with a stable KO of ASCT2 were used for the following studies.

### 4.3. Generation of Syncytin-1-Overexpressing M13SV1 Cells

A vector coding for a Syncytin-1-P2A-PuroR sequence for stable Syncytin-1 overexpression was constructed using the In-Fusion^®^ HD Cloning Kit (Takara, Saint-Germain-en-Laye, France). Briefly, pcDNA3.1(+)_Syncytin-1_Flag plasmid (kind gift of Tiffany R. Frey, Department of Pediatrics, University of Florida, Gainesville, FL, USA; [111]) was linearized by PCR (IF_V2_pcDNA3.1_fwd: 5′-ATC AGC CTC GAC TGT GCC T-3′; IFII_Syn1_rev: 5′-ACT ACT TCC GGC ACT ATT GGG T-3′). P2A-PuroR was amplified by PCR from pcDNA3.1(+)_iCre-T2A-mCherry-P2A-PuroR (IFII_(Syn1)GSG-P2A-PuroR_fwd: 5′-AGT GCC GGA AGT AGT GGA TCT GGA GCA ACA AAC TTC TCA C-3′; IFII_Puro_rev_1: 5′-ACA GTC GAG GCT GAT TTA GGC ACC GGG CTT GC-3′). This fragment with complementary overhangs to the linearized plasmid was ligated into the plasmid in the following In-Fusion reaction, resulting in the vector pcDNA3.1(+)_Syncytin-1-P2A-PuroR. The correctness of the sequence of the plasmid we obtained was verified by Sanger sequencing (Eurofins Genomics, Ebersbach, Germany) using Eurofins Standard Sequencing primers.

### 4.4. Cloning of Dual Split Protein Expression Vectors

The vectors pLV-EF1α-IRES-Hyg_DSP1-7 and pLV-EF1α-IRES-Hyg_DSP8-11 were cloned for the expression of split proteins of the green fluorescent protein (GFP). Therefore, the split protein gene for DSP1-7 was amplified by PCR with the vector Rluc8155-156DSP1-7 (kind gift of Zene Matsuda, University of Tokyo, Japan; RLuc8-155-156-DSP1-7_fwd: 5′-GCT AGG ATC CAT GGC TTC CAA GG-3′; RLuc8-155-156-DSP1-7_rev: 5′-CGA AGC GGC CGC TCT AGA TCA C-3′), creating BamHI and NotI restriction sites. DSP8-11 was amplified by PCR from the vector Rluc8155-156DSP8-11 (kind gift of Zene Matsuda, University of Tokyo, Japan; RLuc8-155-156-DSP8-11_fwd: 5′-AGG AGA TCT ACC ATG CAG AAG AAC GG-3′; RLuc8-155-156-DSP8-11_rev: 5′-GCT CGA AGC GGC CGC TCT AG-3′), creating BglII and NotI restriction sites. The amplificates and the vector pcDNA3.1(+) (Thermo Fisher Scientific, Wesel, Germany) were digested with the respective restriction enzymes and then ligated. Then, the resulting vector pcDNA3.1(+)_DSP1-7 was digested with BamHI and NotI, while pLV-EF1α-IRES-Hyg (kind gift from Tobias Meyer (Addgene plasmid # 85134; http://n2t.net/addgene:85134 (accessed on 21 January 2021); RRID:Addgene_85134)) was digested with BamHI and NotI, and the fragments were ligated to pLV-EF1α-IRES-Hyg_DSP1-7. The other resulting vector pcDNA3.1(+)_DSP8-11 was digested with BglII and NotI, while pLV-EF1α-IRES-Hyg was digested with BamHI and NotI, and the fragments were ligated to pLV-EF1α-IRES-Hyg_DSP8-11. The correctness of the sequence of the plasmid obtained was verified by Sanger sequencing (Eurofins Genomics, Ebersbach, Germany) using Eurofins Standard Sequencing primers.

### 4.5. Cloning of Codon-Optimized Cre Expression Vector

A vector coding for an mCherry-P2A-iCre-P2A-PuroR sequence was constructed using the In-Fusion^®^ HD Cloning Kit (Takara, Saint-Germain-en-Laye, France). Briefly, the pcDNA3.1_mCherry-P2A-Cre-P2A-PuroR plasmid was linearized by PCR (IF_mCherry-iCre-PuroR_VBB_fwd: 5′-ATG ACC GAG TAC AAG CCC ACG-3′; IF_V2_pcDNA3_rev: 5′-CTA GTT AGC CAG AGA GCT CTG CT-3′). The mCherry-P2A fragment was amplified by PCR from pcDNA3.1(+)_mCherry-Cre [17,86] (IF_mCherry_fwd: 5′-TCT CTG GCT AAC TAG ATG GTG AGC AAG GGC GAG GA-3′; IF_mCherry-iCre-PuroR-mCherry_rev: 5′-AGG GCC GGG ATT CTC CTC-3′). The iCre-P2A fragment was amplified by PCR from pcDNA6_mPodocin-Tav-T2A-iCre-T2A-mTurquoise2 (kind gift of Thomas Benzing, University Hospital Cologne, Clinic II for internal medicine, Cologne, Germany; IF_mCherry-iCre-PuroR_iCre_fwd: 5′-TGG AGG AGA ATC CCG GCC CTA TGG TGC CCA AGA AGA AGA GGA A-3′; IF_mCherry-iCre-PuroR_iCre_rev: 5′-GTG GGC TTG TAC TCG GTC ATA GGG CCG GGA TTC TCC TCC ACG TCA CCT GCT TGT TTG AGT AGT GAG AAG TTT GTT GCT CCA GAT CCG TCC CCA TCC TCG AGC AGC-3′). These fragments with complementary overhangs to each other and the linearized plasmid were ligated into the plasmid in the following In-Fusion reaction, resulting in the vector pcDNA3.1(+)_mCherry-P2A-iCre-P2A-PuroR. The correctness of the sequence of the plasmid obtained was verified by Sanger sequencing (Eurofins Genomics, Ebersbach, Germany) using Eurofins Standard Sequencing primers.

### 4.6. Cell Fusion Assays

For the fluorescence double reporter assay, M13SV1 wild-type cells, M13SV1_Syncytin-1 cells and M13SV1_ASCT2KO cells were seeded in a T25 flask and were transfected on the following day with 4 µg Cre expression vector miCP using the jetOPTIMUS^®^ DNA transfection reagent as recommended by the manufacturer (Polyplus, Illkirch, France). Next, 24 h later, the miCP transfected cells as well and the breast cancer cell lines stably expressing the pFDR.1/pFDR.2 vector were set up in a coculture in a ratio of 1:3 in a 96-well plate and in the respective medium of the breast cancer cell line without antibiotics (Table 1). The cells were incubated for 72 h in a humidified atmosphere at 37 °C and 5% CO_2_. Cocultured cells were harvested, washed once with PBS and the amount of green fluorescent cells was quantified by flow cytometry (FACSCalibur, Becton Dickenson, Heidelberg, Germany). Each condition was assayed in triplicates. Freshly harvested cells in the same ratio as the coculture were used as a negative control to adjust the flow cytometer.

For the dual split protein assay, either homotypic cocultures consisting of differently altered M13SV1 cells or heterotypic cocultures with M13SV1 epithelial cells and the breast cancer cell lines were set up. For experiments with homogenic cell fusion, the different M13SV1 cell lines were harvested and the diverse cocultures (Table 2) were set up in a ratio of 1:1 in a 96-well plate. The heterotypic coculture of the differently modified M13SV1 cell lines (DSP8-11) with the three breast cancer cell lines (DSP1-7) was prepared in a ratio of 1:3 in a 96-well plate (Table 2). The medium of the cancer cell line was used as the culture medium and the cells were incubated for 72 h in a humidified atmosphere at 37 °C and 5% CO_2._ Co-cultured cells were harvested, washed once with PBS and the amount of green fluorescent cells was quantified by flow cytometry (FACSCalibur, Becton Dickenson, Heidelberg, Germany). Each condition was assayed in triplicates. Freshly harvested cells in the same ratio as the coculture were used as a negative control to adjust the flow cytometer.

### 4.7. Flow Cytometry

The amount of green fluorescent cells was determined by flow cytometry using a FACSCalibur flow cytometer (Becton Dickenson, Heidelberg, Germany). The cells were harvested, washed with PBS once and resuspended in PBS. The flow cytometry data were analyzed using WinMDI 2.8. 

### 4.8. Western Blot Analysis

Cells were harvested, washed once with PBS, adjusted to a cell number of 2$\;\times 10^{5}$ cells and lysed with 3× Laemmli sample buffer (10 min, 95 °C). The samples were separated by sodium dodecyl sulfate-polyacrylamide gel electrophoresis (SDS-PAGE) in 10% gels and transferred to an Immobilon-P polyvinylidene fluoride (PVDF) membrane (Merck Millipore, Darmstadt, Germany) under semi-dry conditions. The membranes were blocked with 5% (*w*/*v*) non-fat milk powder in Tris-buffered saline with 1% (*v*/*v*) Tween 20 (TBS-T). The following antibodies were used in this study: ASCT2 (clone D7C12, rabbit mAb; Cell Signaling Technology, Leiden, the Netherlands), β-Actin (clone AC-74, mouse mAb; Sigma-Aldrich, Taufkirchen, Germany), Cre recombinase (rabbit pAb; Cell Signaling Technology, Leiden, the Netherlands) and Syncytin-1 (rabbit pAb; Fabgennix, Frisco, TX, USA). Secondary antibodies were coupled to horse radish peroxidase (Cell Signaling Technology, Leiden, the Netherlands). The antibody dilutions used in this study were in accordance with the manufacturer’s instructions. Bands were visualized using Pierce ECL Western blot substrate (Thermo Scientific, Wesel, Germany) and the Aequoria Macroscopic Imaging System (Hamamatsu Photonics Germany, Herrsching am Ammersee, Germany).

### 4.9. Confocal Laser Scanning Microscopy

The fluorescence of co-transfected or fused cells was visualized using confocal laser scanning microscopy (Leica TCS SP5; Leica Microsystems, Wetzlar, Germany). Either 2$\;\times 10^{4}$ cells co-transfected with DSP vectors or a total of 4$\;\times 10^{4}$ cells in the same ratios used for the cell fusion assays were seeded in chamber slides (Nunc Lab-Tek, Thermo Fisher Scientific, Wesel, Germany). After incubation in a humidified atmosphere at 37 °C and 5% CO_2_ for up to 72 h, cells were washed and fixed with 4% paraformaldehyde (PFA; Merck KGaA, Darmstadt, Germany) for 10 min at room temperature, washed with PBS twice and mounted with Fluoromount (Thermo Fisher Scientific, Wesel, Germany). 

### 4.10. Live Cell Imaging

The coculturing of M13SV1_Syn1_miCP cells with MDA-MB-435S_pFDR.1 cells was recorded every 30 min with the help of the Incucyte ^®^ SX5 (Sartorius Lab Instruments GmbH & Co. KG, Göttingen, Germany). Two hours after setting up the coculture, the plate was transferred to the device and the recording was carried out over about 40 h. The time-lapse video, showing the fluorescence switch of the fused cells, can be found in the Supplementary Materials Video S1.

### 4.11. Statistical Analysis

Statistical analysis was performed using GraphPad PRISM 9.5 (https://graphpad.com (accessed on 6 December 2022)). A detailed description of which statistical test was used is given in the appropriate figure legends.

## Figures and Tables

**Table 1:** Cocultures set up for measurement with FDR assay.

Type of Merger	Cells in Coculture		
heterotypic	M13SV1_miCP	x	HS578T_pFDR.2 MDA-MB-231_pFDR.2 MDA-MB-435S_pFDR.1
	M13SV1_ASCT2KO_miCP	x	HS578T_pFDR.2 MDA-MB-231_pFDR.2 MDA-MB-435S_pFDR.1
	M13SV1_Syn1_miCP	x	HS578T_pFDR.2 MDA-MB-231_pFDR.2 MDA-MB-435S_pFDR.1

**Table 2:** Cocultures set up for measurement with DSP assay.

Type of Merger	Cells in Coculture		
homotypic	M13SV1_DSP1-7	x	M13SV1_DSP8-11 M13SV1_ASCT2KO_DSP8-11 M13SV1_Syn1_DSP8-11
	M13SV1_ASCT2KO_DSP1-7	x	M13SV1_ASCT2KO_DSP8-11
	M13SV1_Syn1_DSP1-7	x	M13SV1_Syn1_DSP8-11 M13SV1_ASCT2KO_DSP8-11
heterotypic	M13SV1_miCP	x	HS578T_pFDR.2 MDA-MB-231_pFDR.2 MDA-MB-435S_pFDR.1
	M13SV1_ASCT2KO_miCP	x	HS578T_pFDR.2 MDA-MB-231_pFDR.2 MDA-MB-435S_pFDR.1
	M13SV1_Syn1_miCP	x	HS578T_pFDR.2 MDA-MB-231_pFDR.2 MDA-MB-435S_pFDR.1

## Data Availability

All data will be shared upon reasonable request.

## Supplementary Materials

The following supporting information can be downloaded at: https://www.mdpi.com/article/10.3390/ijms25115668/s1.

## Abbreviations

ASCT2	alanine serine cysteine transporter 2
BSA	bovine serum albumin
CS/ICs	cancer stem/initiating cells
DSP	dual split protein
FDR	fluorescence double reporter
GFP	green fluorescent protein
HERV	human endogenous retrovirus
KO	knock-out
PVDF	polyvinylidene fluoride
SDS-PAGE	sodium dodecyl sulfate-polyacrylamide gel electrophoresis
Syn1	Syncytin-1
TBS-T	Tris-buffered saline with 1% (*v*/*v*) Tween 20
